# Smad4 is required to inhibit osteoclastogenesis and maintain bone mass

**DOI:** 10.1038/srep35221

**Published:** 2016-10-12

**Authors:** Mayu Morita, Shigeyuki Yoshida, Ryotaro Iwasaki, Tetsuro Yasui, Yuiko Sato, Tami Kobayashi, Ryuichi Watanabe, Takatsugu Oike, Kana Miyamoto, Masamichi Takami, Keiko Ozato, Chu-Xia Deng, Hiroyuki Aburatani, Sakae Tanaka, Akihiko Yoshimura, Yoshiaki Toyama, Morio Matsumoto, Masaya Nakamura, Hiromasa Kawana, Taneaki Nakagawa, Takeshi Miyamoto

**Affiliations:** 1 Division of Oral and Maxillofacial surgery, Department of Dentistry and Oral Surgery, Keio University School of Medicine, 35 Shinano-machi, Shinjuku-ku, Tokyo 160-8582, Japan.; 2 Department of Orthopedic Surgery, Faculty of Medicine, The University of Tokyo, 7-3-1 Hongo, Bunkyo-ku, Tokyo 113-8859, Japan; 3 Department of Orthopedic Surgery, Keio University School of Medicine, 35 Shinano-machi, Shinjuku-ku, Tokyo 160-8582, Japan; 4 Department of Musculoskeletal Reconstruction and Regeneration Surgery, Keio University School of Medicine, 35 Shinano-machi, Shinjuku-ku, Tokyo 160-8582, Japan; 5 Department of Advanced Therapy for Musculoskeletal Disorders, Keio University School of Medicine, 35 Shinano-machi, Shinjuku-ku, Tokyo 160-8582, Japan; 6 Department of Pharmacology, Showa University School of Dentistry, 1-5-8 Hatanodai, Sinagawa-ku, Tokyo 142-0033, Japan; 7 Laboratory of Molecular Growth Regulation, Genomics of Differentiation Program, NICHD, National Institutes of Health, Bethesda, MD; 8 Faculty of Health Sciences, University of Macau, Macau SAR, China; 9 Genome Science Division, Research Center for Advanced Science and Technology (RCAST), The University of Tokyo, 4-6-1 Komaba, Meguro-ku, Tokyo 153–8904, Japan; 10 Department of Microbiology and Immunology, Keio University School of Medicine, 35 Shinano-machi, Shinjuku-ku, Tokyo 160-8582, Japan

## Abstract

Bone homeostasis is maintained as a delicate balance between bone-resorption and bone-formation, which are coupled to maintain appropriate bone mass. A critical question is how bone-resorption is terminated to allow bone-formation to occur. Here, we show that TGFβs inhibit osteoclastogenesis and maintain bone-mass through Smad4 activity in osteoclasts. We found that latent-TGFβ1 was activated by osteoclasts to inhibit osteoclastogenesis. Osteoclast-specific *Smad4* conditional knockout mice (Smad4-cKO) exhibited significantly reduced bone-mass and elevated osteoclast formation relative to controls. TGFβ1-activation induced expression of *Irf8* and *Bcl6*, both of which encode factors inhibiting osteoclastogenesis, by blocking their negative regulator, *Prdm1*, in osteoclasts in a Smad4-dependent manner. Reduced bone-mass and accelerated osteoclastogenesis seen in Smad4-cKO were abrogated by *Prdm1* deletion. Administration of latent-TGFβ1-Fc to wild-type mice antagonized LPS-induced bone destruction in a model of activated osteoclast-mediated bone destruction. Thus, latent-TGFβ1-Fc could serve as a promising new therapeutic agent in bone diseases marked by excessive resorption.

Bone is continuously resorbed and constructed, and bone volume is controlled as a balance of both activities, a process termed “coupling”^1^. The coupling system is also critical to determine regions for new bone production, which are often sites where resorption has occurred^2^. Coupling failure results in bone diseases such as osteoporosis, in which bone formation following resorption is less effective, reducing bone mass^3^. To date, drugs such as bisphosphonates have been launched as osteoporosis therapy; however, their use frequently causes multiple adverse effects, including osteonecrosis of the jaw due to excessive inhibition of osteoclastic activity beyond levels required for physiological bone turnover^4^. Thus, understanding the coupling system is required to better enable us to increase bone formation at required levels and at sites where resorption occurs.

Several factors have been proposed to function as coupling factors^5^. For example, osteoclast/osteoblast signaling is reportedly transduced by ephrin B2/EphB4, Ephrin A2/EphA2, and Semaphorin 4D/PlexinB1 interactions or by Semaphorin 3A secreted by osteoblasts^6^,^7^,^8^,^9^. In addition, mice deficient in genes encoding either dendritic cell-specific transmembrane protein or the v-ATPase V0 subunit d2 exhibit increased osteoblastic activity, reduced osteoclastic bone resorption, and increased bone bone mass^10^,^11^. Collagen triple helix repeat containing 1 (Cthrc1) produced by bone-resorbing mature osteoclasts is also reportedly required for bone formation^12^.

Transforming growth factor beta 1 (TGFβ1), a member of the TGFβ superfamily, also serves as a coupling factor. This ligand family consists of TGFβ1–5 as well as bone morphogenetic proteins (BMPs), however, TGFβ4 and TGFβ5 are expressed only in chick and *Xenopus*, respectively^13^,^14^,^15^,^16^,^17^. TGFβ ligands signal by binding to specific receptors and activating receptor-regulated transcription factors called Smads (R-Smads), namely Smad2/3 (for TGFβ1–5) and Smad1/5/8 (for BMPs), which form oligomers with the common mediator Smad4. Thus, loss of Smad4 results in loss of both TGFβs-Smad2/3 and BMPs-Smad1/5/8 signals^18^. Osteoblast-specific Smad4-deficient mice show altered bone formation^19^, while chondrocyte-specific Smad4-deficient mice reportedly exhibit dwarfism^20^; however, although bone tissues comprised of osteoclasts are rich in TGFβs and BMPs^21^,^22^, Smad4 function in those cells is largely unknown.

TGFβ1 and insulin like growth factor 1 (IGF1) reportedly accumulate in bone matrix and are released following osteoclastic bone resorption, stimulating osteoblastic bone formation^23^,^24^. TGFβ1 is initially produced in an inactive form (latent-TGFβ1), which is then activated extracellularly under highly acidic conditions^25^. Activated TGFβ1 reportedly promotes bone marrow stromal cell migration to enable bone formation^25^. Nonetheless, TGFβ1 function in osteoclasts is controversial: various investigators report that TGFβ1 exerts both stimulatory and inhibitory effects on osteoclast differentiation *in vitro*, the former of which was reported by majority as TGFβ1 action on osteoclasts^26^,^27^,^28^. Recently, osteoclast-specific ablation of TGFβ receptor 2 in mice was reportedly resulted in no significant impact on osteoclast numbers or activity *in vivo*^29^. BMP2 reportedly stimulates osteoclastogenesis^30^,^31^; however, osteoclast-specific conditional ablation of the gene encoding its receptor BMP receptor type 1A (BMPR1a) increases osteoclast differentiation *in vivo*^32^.

Osteoclast differentiation is regulated as a balance between stimulators and inhibitors^33^,^34^. RANKL-dependent factors that promote differentiation include Nuclear factor of activated T cells 1 (NFATc1), c-Fos and Blimp1, the latter encoded by the *PR domain 1* (*Prdm1*)^35^,^36^,^37^,^38^. In contrast, interferon regulatory factor 8 (Irf8) and B cell lymphoma 6 (Bcl6) reportedly inhibit osteoclast differentiation, and are suppressed by RANKL stimulation in osteoclasts via Blimp1 activity^38^,^39^,^40^.

Here, we show that Smad4 is expressed in osteoclasts and report that osteoclast-specific Smad4 conditional knockout mice (Smad4-cKO: *Cathepsin K* (*Ctsk)*^*Cre/*+^/*Smad4*^*flox/flox*^) exhibit significantly reduced bone mass due to accelerated osteoclast formation. High TGFβ1 concentrations inhibited osteoclast differentiation of wild-type cells *in vitro*, and such inhibition was blocked in Smad4 cKO cells. We also show that TGFβ1 inhibits *Prdm1* expression, which in turn upregulates *Irf8* and *Bcl6* expression, inhibiting osteoclast differentiation. Reduced bone mass and elevated osteoclastogenesis in Smad4-cKO were abrogated in Smad4/Blimp1 doubly mutant mice. Latent-TGFβ1 was converted to an active form by osteoclastic activity in cultured cells, and administration of latent-TGFβ1-Fc to wild-type mice blocked LPS-induced bone destruction. We conclude that following bone resorption, inhibition of osteoclastogenesis by activated TGFβ1 via Smad4 expressed in osteoclasts is crucial to maintain bone mass.

## Results

### Osteoclastogenesis is differentially regulated in osteoclast progenitor cells by high concentrations of TGFβ1 or TGFβ3 *in vitro*

**Figure 1:**
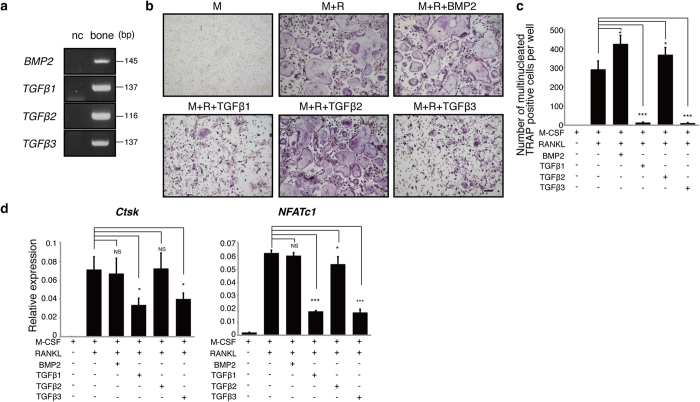
TGFβ1/β3 and BMP2/TGFβ2 have differential effects on osteoclastogenesis. (**a**) Expression of TGFβ1–3 and BMP2 transcripts in mouse humerus bone was confirmed. Osteoclast progenitors from wild-type mice were cultured with recombinant BMP2 (200 ng/ml), TGFβ1 (10 ng/ml), TGFβ2 (10 ng/ml) or TGFβ3 (10 ng/ml) in the presence of M-CSF (50 ng/ml; M) and RANKL (25 ng/ml; R) for five days and then assessed for osteoclast formation by TRAP staining (**b**), by counting the number of multi-nuclear TRAP-positive cells (**c**) and by expression of the osteoclast markers *Ctsk* and *NFATc1* based on realtime PCR (**d**). Data represent mean *Ctsk* or *NFATc1* expression relative to *β-actin* ± SD (*n* = 3). Bar = 100 μm. *P < 0.05; ***P < 0.001; NS, not significant. Representative data of at least three independent experiments are shown.

To assess expression of TGFβ factors in bone, we undertook analysis of transcripts encoding these factors in bone tissues of wild-type mice and identified TGFβ1, 2, 3 and BMP2 mRNAs (Fig. 1a). We also found that osteoclastogenesis, as assessed by expression of osteoclastic genes such as *Cathepsin K* (*Ctsk*) and *NFATc1* following RANKL treatment of cultured Raw264.7 cells, was enhanced by co-incubation of cells with RANKL plus either TGFβ1 or β3 (Supplementary Fig. 1). Osteoclast differentiation in bone marrow macrophages (BMMs) was also significantly inhibited by SB431542, a TGFβ inhibitor, *in vitro* (Supplementary Fig. 2a–c). Interestingly, *in vitro* osteoclastogenesis in wild-type BMMs was stimulated at a lower concentration (0.016 ng/ml) of either TGFβ1 or TGFβ3, while differentiation was significantly inhibited at higher concentrations (0.4, 2 or 10 ng/ml) of either TGFβ1 or TGFβ3 dose-dependently (Fig. 1b–d, Supplementary Fig. 3 and Supplementary Fig. 4). In contrast, osteoclast differentiation from wild-type BMMs was stimulated by high concentrations of either TGFβ2 (10 ng/ml) or BMP2 (200 ng/ml) (Fig. 1b,c). Osteoclastogenesis, as evidenced by appearance of multi-nuclear TRAP-positive cells, was stimulated by either 40 or 200 ng/ml BMP2 but inhibited by 1,000 ng/ml of BMP2 (Supplementary Fig. 5). These results suggest that osteoclastogenesis is regulated in a complex manner by TGFβ superfamily members in the bone microenvironment.

### Smad4 is required to inhibit osteoclastogenesis and maintain bone mass

**Figure 2:**
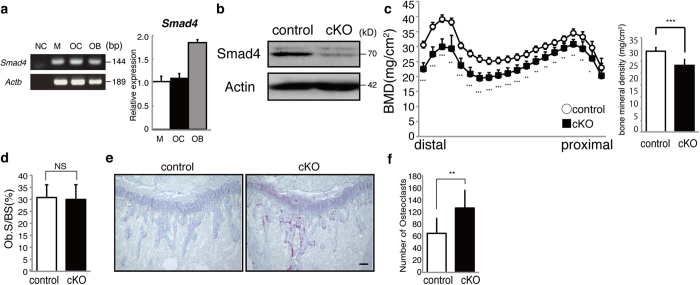
Smad4 is expressed in osteoclasts and required for osteoclast inhibition. (**a**) Electrophoresis gel images of RT-PCR (left panels) or quantitative real-time PCR (right panel) analysis of *Smad4* expression in macrophages (M), osteoclasts (OC) and osteoblasts (OB). *β-actin* served as an internal control. Data represent mean *Smad4* expression relative to *β-actin* ± SD. (**b**) Western blotting in osteoclasts to assess Smad4 deletion efficiency in Smad4 cKO (cKO) compared with control (*Smad4*^*flox/flox*^) osteoclasts. (**c**) Bone mineral density (BMD) of femurs bisected equally longitudinally from control (*Smad4*^*flox/flox*^) and Smad4-cKO (cKO) mice. *P < 0.05; **P < 0.01; ***P < 0.001. (**d**) Bone histomorphometrical analysis of femurs from control and Smad4 cKO (cKO) female mice. Osteoblast surface per bone surface (Ob.S/BS) was determined. NS, not significant. Tibial sections from control (*Smad4*^*flox/flox or flox/*+^) and Smad4 cKO mice were stained with TRAP (**e**), and the number of TRAP-positive osteoclasts was scored (**f**). Bar = 100 μm. **P < 0.01. Representative data of at least two independent experiments are shown.

As noted, lack of Smad4 results in abrogation of both TGFβ and BMP signaling. We detected *Smad4* expression in osteoclasts (Fig. 2a). To assess roles of Smad4 and downstream signaling in regulating osteoclastogenesis and bone mass *in vivo*, we generated osteoclast-specific Smad4 conditional knockout mice (Smad4 cKO) using *Ctsk*-Cre mice (Fig. 2b). Based on DEXA analysis, Smad4 cKO mice exhibited significantly reduced bone mass with accelerated osteoclastogenesis as analyzed by TRAP staining and bone morphometric analysis compared with controls *in vivo* (Fig. 2c–f). Osteoblastogenesis was normal in Smad4 cKO mice, while osteoclast formation was activated (Fig. 2d–f). Thus, reduced bone mass seen in Smad4 cKO mice is likely due to elevated osteoclastogenesis *in vivo*.

### TGFβ1 and β3 inhibit osteoclast differentiation via Smad4

**Figure 3:**
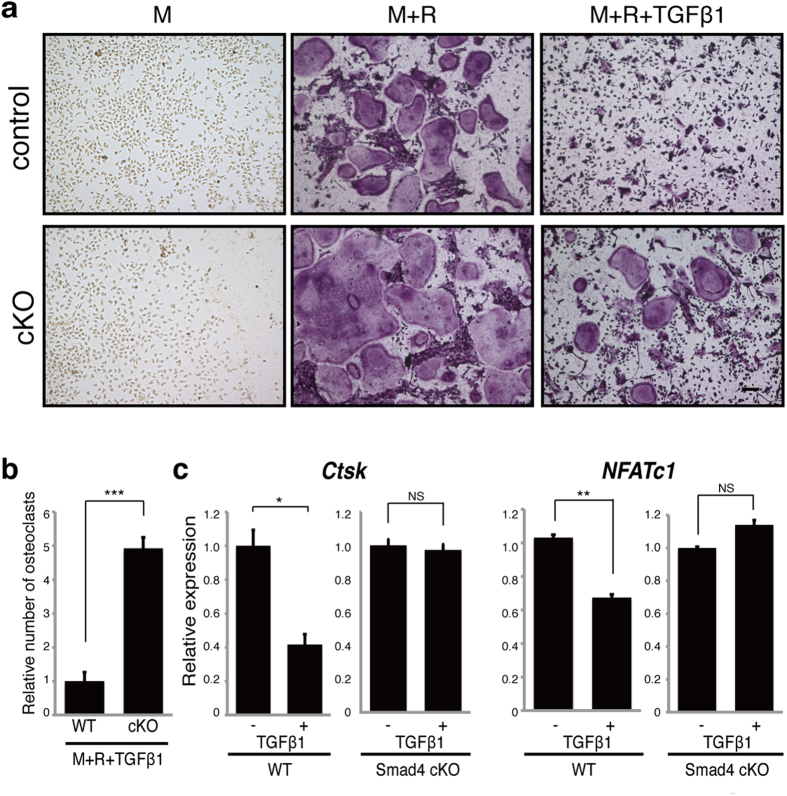
High TGFβ1 concentrations inhibit osteoclastogenesis via Smad4 activity. Osteoclast progenitors from control (*Smad4*^*flox/flox or flox/*+^) or Smad4-cKO (cKO) mice were cultured with or without 10 ng/ml TGFβ1 in the presence or absence of M-CSF (M) and RANKL (R). Osteoclast formation was evaluated by TRAP staining (**a**), by the number of multi-nuclear TRAP-positive cells (**b**) and by *Ctsk* and *NFATc1* expression as analyzed by realtime PCR (**c**). Data represent mean *Ctsk* or *NFATc1* expression relative to *β-actin* ± SD (*n* = 3). Bar = 100 μm. *P < 0.05; **P < 0.01; ***P < 0.001; NS, not significant. Representative data of at least three independent experiments are shown.

We next focused on identifying osteoclast-inhibiting signals mediated by Smad4 *in vitro* (Fig. 3). Osteoclastogenesis in wild-type BMMs as analyzed by formation of multi-nuclear TRAP-positive cells *in vitro*, was significantly inhibited in the presence of high concentrations of TGFβ1 or TGFβ3, and inhibition was significantly reversed in Smad4 cKO cells (Fig. 3a,b and Supplementary Fig. 6a,b). Expression of the osteoclast differentiation markers *Ctsk* and *NFATc1* was significantly inhibited by treatment of wild-type osteoclasts with either TGFβ1 or TGFβ3 *in vitro*, an effect reversed in Smad4 cKO cells (Fig. 3c and Supplementary Fig. 6c).

### Smad4 regulates *Bcl6* and *Irf8* expression

**Figure 4:**
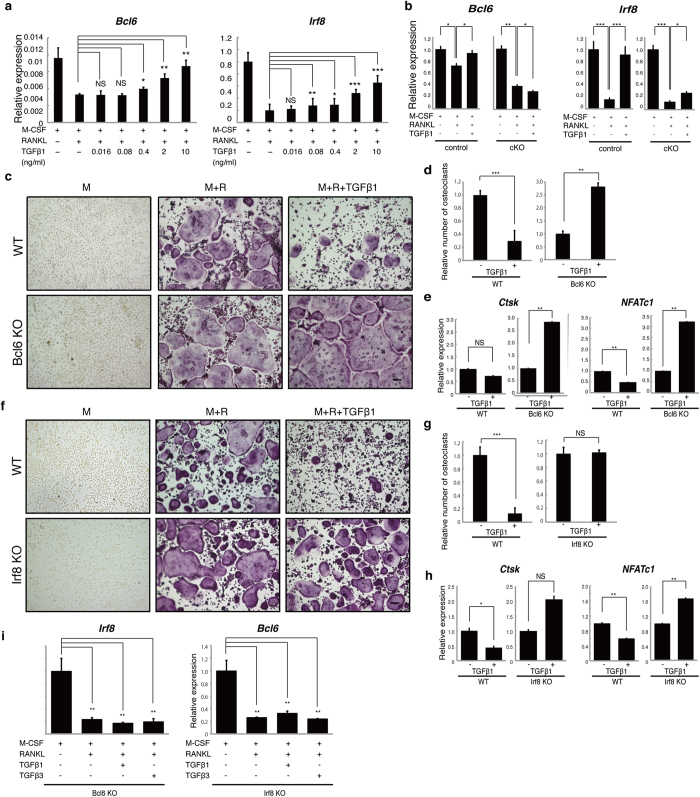
TGFβ1 stimulates increases in *Bcl6* and *Irf8* expression in osteoclasts via Smad4 expressed. (**a**) Osteoclast progenitors were isolated from wild-type mice and cultured in the presence or absence of M-CSF (M) and RANKL (R) with or without indicated concentrations of TGFβ1. *Irf8* and *Bcl6* expression was then determined by realtime PCR. Data represent mean *Bcl6* or *Irf8* expression relative to *β-actin* ± SD (*n* = 3). (**b**) Osteoclast progenitors were isolated from control (*Smad4*^*flox/flox or flox/*+^) or Smad4 cKO (cKO) mice and cultured in the presence or absence of RANKL with or without 10 ng/ml of TGFβ1. *Irf8* and *Bcl6* expression was determined by realtime PCR. Data represent mean *Bcl6* or *Irf8* expression relative to *β-actin* ± SD (*n* = 3). (**c–h**) Osteoclast progenitors were isolated from wild-type, Bcl6-deficient (**c*****–*****e**) or Irf8-deficient (**f–h**) mice and cultured in the presence or absence of M-CSF (M) and RANKL (R) with or without 10 ng/ml TGFβ1. Osteoclast formation was evaluated by TRAP staining (**c**,**f**), by the number of multi-nuclear TRAP-positive cells (**d**,**g**) and by *Ctsk* and *NFATc1* expression as analyzed by realtime PCR (**e**,**h**). *Irf8* and *Bcl6* expression was determined in Bcl6 and Irf8-deficient mice, respectively, by realtime PCR (**i**). Data represent mean *Ctsk* or *NFATc1* or expression relative to *β-actin* ± SD (*n* = 3). Bar = 100 μm. *P < 0.05; **P < 0.01; ***P < 0.001; NS, not significant. Representative data of at least two independent experiments are shown.

To define molecular mechanisms underlying TGFβ inhibition of osteoclastogenesis through Smad4, we analyzed expression of potential inhibitory factors following treatment of wild-type osteoclasts with TGFβs. Candidates included *Bcl6* and *Irf8*, both transcriptional repressors and reported inhibitors of osteoclastogenesis^39^,^40^. *Bcl6* and *Irf8* mRNA expression was upregulated following stimulation of wild-type osteoclasts with TGFβ1 (Fig. 4a). Interestingly, *Bcl6* and *Irf8* upregulation was significantly blocked in Smad4 cKO cells (Fig. 4b), suggesting that such upregulation is dependent on Smad4. Thus, next we treated Bcl6-deficient BMMs with TGFβ1 or β3 and found that their inhibition of osteoclast formation was abrogated relative to wild-type cells (Fig. 4c,d, and Supplementary Fig. 7a,b). Likewise, decreased expression of the osteoclastic genes *Ctsk* and *NFATc1* seen following TGFβ1 or β3 treatment in wild-type osteoclasts was significantly rescued in Bcl6-deficient osteoclasts (Fig. 4e, Supplementary Fig. 7c). Similarly, Irf8-deficient cells were resistant to inhibition of osteoclastogenesis and suppression of osteoclastic gene expression by either TGFβ1 or β3 (Fig. 4f–h, Supplementary Fig. 7d–f).

Interestingly, deficiency of either Bcl6 or Irf8 was sufficient to block TGFβ1- or β3-induced inhibition of osteoclastogenesis (Fig. 4a–h), and *Irf8* or *Bcl6* expression was significantly inhibited in Bcl6- or Irf8-deficient osteoclasts, respectively (Fig. 4i).

### Blimp1 is a direct target of Smad4 in osteoclasts

**Figure 5:**
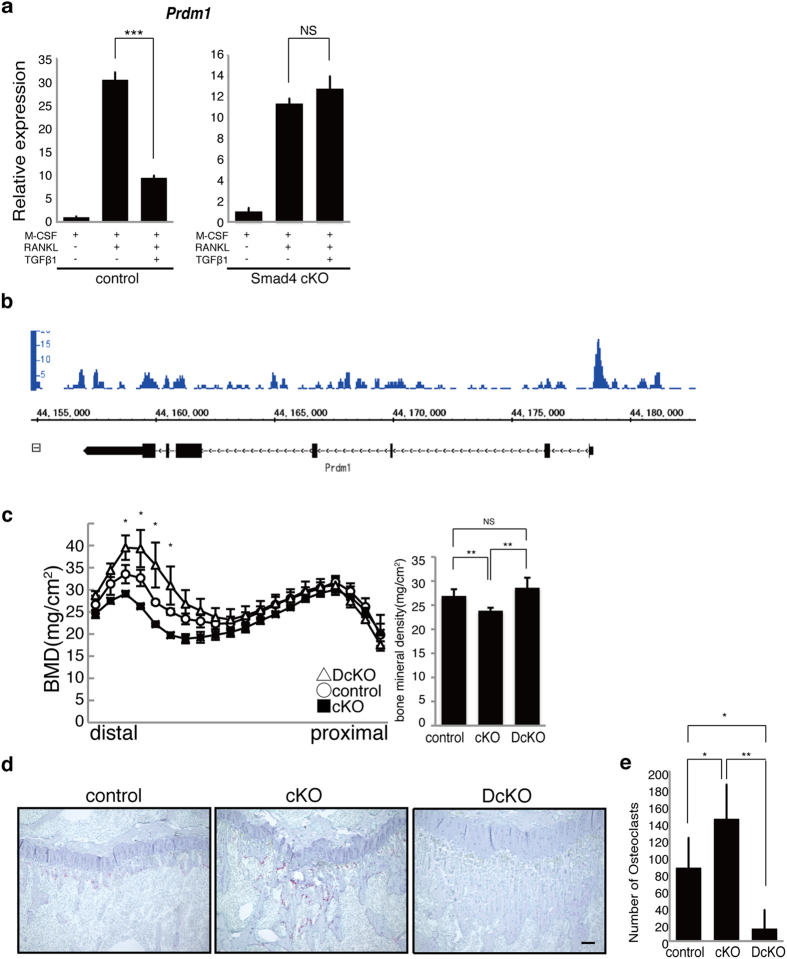
*Prdm1* deletion rescues bone loss in Smad4-cKO mice. (**a**) Osteoclast progenitors from control (*Smad4*^*flox/flox or flox/*+^) or Smad4 cKO mice were cultured with or without 10 ng/ml TGFβ1 in the presence or absence of M-CSF and RANKL. *Prdm1* expression was analyzed by realtime PCR. Data represent mean *Prdm1* expression relative to *β-actin* ± SD (*n* = 3). (**b**) Osteclast progenitor cells from wild-type mice were cultured in the presence of M-CSF and RANKL with 10 ng/ml TGFβ1, and chromatin immune precipitation-sequencing analysis was preformed by using anti-Smad2/3 antibody. Smad2/3 bound to regions upstream of the *Prdm1* gene. (**c**) Bone mineral density of femurs divided equally longitudinally from control, Smad4 cKO and *Smad4*/*Prdm1* double-mutant (DcKO) female mice. (**d**,**e**) Tibial sections from control (*Smad4*^*flox/flox or flox/*+^), Smad4 cKO and DcKO mice were stained with TRAP (**d**), and the number of TRAP-positive osteoclasts was scored (**e**). Data represent mean number of TRAP-positive cells per section ± SD (*n* = 3). Bar = 100 μm. *P < 0.05; **P < 0.01; NS, not significant. Representative data of at least two independent experiments are shown.

Both *Bcl6* and *Irf8* expression in osteoclasts is reportedly negatively regulated by Blimp1, a transcriptional repressor encoded by *Prdm1*^39^,^40^. Thus, we asked whether elevated *Bcl6* and *Irf8* expression seen following TGFβ1 or TGFβ3 treatment was accompanied by decreased *Prdm1* expression. In accordance, *Prdm1* mRNA expression was significantly inhibited by either TGFβ1 or TGFβ3 treatment of wild-type osteoclasts (Fig. 5a, Supplementary Fig. 8), but such *Prdm1* inhibition was abrogated in Smad4 cKO cells (Fig. 5a, Supplementary Fig. 8). To assess whether *Prdm1* is a direct target of Smad in osteoclasts, we employed chromatin immune precipitation sequencing (ChIP seq) analysis using anti-Smad2/3 antibodies, and observed that Smad2/3 bound to an upstream region of the *Prdm1* gene in osteoclasts under TGFβ1 stimulation (Fig. 5b). When we generated osteoclast-specific *Smad4*/*Prdm1* double knockout (DcKO: *Ctsk*^*Cre*/+^/*Smad4*^*f*/*f*^*Prdm1*^*f*/*f*^) mice, in which *Prdm1* is deleted from Smad4 cKO mice, we found that the significantly decreased bone mass seen in Smad4 cKO mice was reversed and rather increased in DcKO mice (Fig. 5c–e). These observations suggest that Smad4 is required for *Prdm1* inhibition in osteoclasts and to maintain bone mass following stimulation with either TGFβ1 or TGFβ3.

We also found that *Prdm1* expression was significantly upregulated by either TGFβ1 or TGFβ3 in Raw263.7 cells (Supplementary Fig. 9). Although, *Bcl6* expression was rather upregulated, elevated *Prdm1* expression may explain, at least in part, why osteoclastogenesis was stimulated in Raw264.7 cells by either TGFβ1 or TGFβ3. Osteoclast formation was stimulated by 200 ng/ml of BMP2, however, either *Prdm1, Bcl6* or *Irf8* expression level remained unchanged following BMP2 treatment of wild-type osteoclasts (Supplementary Fig. 10). Furthermore, *Prdm1* expression was significantly inhibited, while *Bcl6* and *Irf8* expression was significantly upregulated in SB431542-treated wild-type osteoclasts (Supplementary Fig. 11).

### Latent-TGFβ1 inhibits LPS-induced osteoclast formation and bone destruction

**Figure 6:**
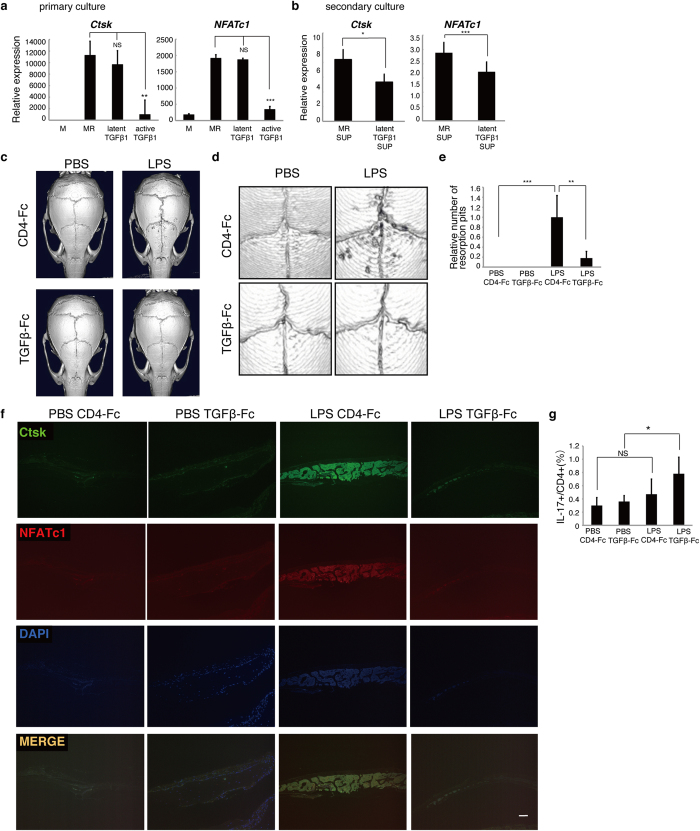
Latent TGFβ1 is converted to an active form by osteoclastic activity. (**a**) Osteoclast progenitors from wild-type mice were cultured in the presence of M-CSF and RANKL with or without either active- or latent-TGFβ1 (10 ng/ml each) for primary culture. Quantitative real-time PCR analysis of osteoclastic mRNAs was then undertaken. Data represent mean *Ctsk* or *NFATc1* expression relative to *β-actin* ± SD (*n* = 3). (**b**) Primary culture supernatants were collected from wild-type cells and transferred to secondary cultures of wild-type osteoclast progenitors, which were then treated with M-CSF and RANKL, and *Ctsk* and *NFATc1* expression was analyzed by realtime PCR. Data represent mean *Ctsk* or *NFATc1* expression relative to *β-actin* ± SD (*n* = 3). (**c–g**) LPS (50 mg/kg) was administered subcutaneously onto the skull of living 8-week-old female wild-type mice with or without 16 mg of latent-TGFβ1. Five days later, osteolysis in calvariae was analyzed by μCT (**c**, low magnification; **d**, high magnification). PBS injection served as a negative control. The number of resorption pits per calvariae was scored. (**e**). Data represent mean resorption pit number per calvariae ± SD (*n* = 5). Sections were stained with mouse anti-Ctsk and rabbit anti-NFATc1 antibodies, followed by Alexa488-conjugated goat anti-mouse Ig’ antibody, Alexa488-conjugated goat anti-rabbit Ig’ antibody and DAPI. Sections were then observed by fluorescence microscopy (**f**). Spleen cells were stained with anti-CD4 and anti-IL-17 antibodies, and the frequency of TH17 cells (CD4^+^IL-17^+^ cells) was analyzed by flow cytometry (**g**). Data represent mean TH17 cell frequency ± SD (*n* = 5). Bar = 100 μm. *P < 0.05; NS, not significant. Representative data of at least two independent experiments are shown.

As reported, TGFβ1 is converted from a non-active, latent-TGFβ1 form to an activated form^25^. First, we established primary cultures of wild-type osteoclasts with or without latent-TGFβ1, and found that osteoclastogenesis *in vitro* was inhibited when wild-type BMMs were treated with active TGFβ1 but not by latent-TGFβ1 (Fig. 6a). Then, we treated wild-type BMMs with supernatants from primary cultures in the presence of M-CSF and RANKL (Fig. 6b). Latent-TGFβ1 is reportedly activated by osteoclastic bone-resorption^25^. In accordance, we found that osteoclastogenesis was inhibited in secondary cultures treated with supernatants from osteoclasts cultured with latent-TGFβ1 (Fig. 6b). Based on these results, we concluded that administered latent-TGFβ1 is converted to an active form by osteoclast to inhibit osteoclast formation. To test this hypothesis, we administered latent-TGFβ1-Fc or control CD4-Fc protein by injection *in vivo* in a mouse model of LPS-induced bone destruction, in which LPS was injected on wild-type mouse calvariae. We found that LPS-induced bone-resorption and osteoclast formation as analyzed by micro CT (μCT), and anti-Ctsk with anti-NFATc1 staining, respectively, were significantly inhibited by latent-TGFβ1-Fc compared with CD4-Fc administration (Fig. 6c–f). TGFβ1 signaling is known to promote differentiation of TH17 cells, a type of osteoclastogenic T cells implicated in bone destruction^41^,^42^. Indeed, in an LPS-induced model of bone destruction, we found that TH17 cell frequency significantly increased in mice treated with LPS together with latent-TGFβ1-Fc compared with control mice treated with PBS plus latent-TGFβ1-Fc (Fig. 6g). The fact that bone destruction was inhibited by latent-TGFβ1-Fc, even under elevated TH17 cell conditions, suggests that latent-TGFβ1-Fc could antagonize bone destruction in osteoclast-activating conditions.

## Discussion

Numerous bone-regulating factors maintain bone homeostasis^1^,^43^. Among them, factors activating signals via Smad4, including TGFβ and BMP, reportedly support osteoblastic cell migration, proliferation, differentiation and bone formation *in vivo* and *in vitro* (Supplementary Fig. 12a)^19^,^44^. This study demonstrates that Smad4 mediates osteoprotective signals that are coupled with osteoclastic bone resorption and acts as part of a negative feedback mechanism (Supplementary Fig. 12b,c). Our findings suggest overall that Smad4 plays a role in both inhibiting bone resorption and activating bone formation (Supplementary Fig. 12). Here, we show that latent-TGFβ is activated by osteoclasts, which inhibits their activity (Supplementary Fig. 12b).

The activity of TGFβ superfamily members in osteoclasts reportedly varies^26^,^27^,^28^,^29^,^32^, and we show that TGFβ1/β3 inhibits osteoclastogenesis, while TGFβ2/BMP2 stimulates it. However, the significant reduction in bone mass and elevated osteoclast formation we report here in Smad4 cKO mice suggests that in this system inhibitory signals via Smad4 are dominant over stimulators. Since Smad4 null mice exhibit embryonic lethality^45^, Smad4 function in osteoclasts and bones has not previously been characterized. The Cre/loxP system employed here did not completely abrogate Smad4 activity in osteoclasts, and some Smad4 function may remain. Nonetheless, it allowed us evaluate Smad4 function in osteoclastogenesis and bone at adult stages. Those signals via TGFβ result from conversion of latent-TGFβ to TGFβ1, which in turn blocks expression of *Prdm1*, a repressor of osteoclastogenesis. Loss of the repressor encoded by *Prdm1* upregulates *Bcl6* and *Irf8*, both of which repress osteoclast differentiation (Supplementary Fig. 12c). Although, at present, molecular mechanisms underlying are not clear, we found that *Irf8* or *Bcl6* expression was significantly inhibited in Bcl6- or Irf8-deficient osteoclasts, respectively (Fig. 4i), suggesting that these factors regulate each other in osteoclasts.

TGFβ and BMP signaling is regulated in a complex manner in osteoblasts^44^. Indeed, TGFβ1 is reportedly required for osteoblastogenesis^19^,^44^, while it is also reported to inhibit osteoblastogenesis induced by BMP2^46^. However, there is net decrease in bone mass seen in osteoblast-specific Smad4-deficient mice^19^, suggesting that Smad4 signals in osteoblasts positively regulate bone formation. Thus overall, although why high concentration of BMP2 (1,000 ng/ml) inhibited osteoclast formation was not clear, Smad4 signaling in both osteoclasts and osteoblasts results in increases in bone mass.

Recent advances in developing anti-osteoporosis drugs have resulted in both anti-resorptive agents such as bisphosphonate or anti-RANKL antibodies, and bone-forming drugs, such as teriparatide^47^,^48^,^49^,^50^. Both types have significant therapeutic effects in increasing bone mass and preventing fractures in osteoporosis patients^51^. However, the broad effects of anti-resorptive or bone-forming agents in inhibiting or promoting osteoclast differentiation/function, respectively, can cause adverse side effects such as jaw osteonecrosis, super suppressive bone turnover or osteosarcoma formation^4^,^52^. As alternatives, investigators are currently seeking novel reagents targeting specific sites where bone formation is required following resorption. Our data strongly suggests that the TGFβ/Smad4 system is specifically activated at such sites. Our observations therefore provide a molecular basis for developing agents that both inhibit bone-resorption and activate bone-formation.

## Methods

### Mice

Wild-type mice were purchased from Sankyo Labo Service (Tokyo, Japan). *Ctsk*^*cre/*+^, *Smad4*^*f/f*^, *Prdm1*^*f/f*^, Bcl6-deficient and Irf8-deficient mice were prepared as previously described^39^,^40^,^53^,^54^. Animals were maintained under specific pathogen-free conditions in animal facilities certified by the Keio University Institutional Animal Care and Use Committee, and animal protocols were approved by that committee. All animal studies were performed in accordance with the Guidelines of the Keio University animal care committee.

### Analysis of skeletal morphology

*Ctsk*^*cre/*+^*Smad4*^*f/f*^, *Ctsk*^*cre/*+^*Smad4*^*f/f*^*Prdm1*^*f/f*^ and control littermates were necropsied, and their hind limbs were removed, fixed in 70% ethanol, and subjected to DEXA analysis to measure bone mineral density, and analysis of bone histomorphometric parameters. Bones were collected from 8-week-old female mice.

### *In vitro* osteoclast formation

For *in vitro* analysis, bone marrow cells isolated from femurs and tibias were cultured for 72 h in MEM (Sigma-Aldrich Co.) containing 10% (vol/vol) heat-inactivated FBS (JRH Biosciences) and GlutaMax (Invitrogen Corp.) supplemented with M-CSF (50 ng/mL, Kyowa Hakko Kirin Co.). Subsequently, adherent cells were collected and cultured in 96-well plates (1 × 10^5^ cells per well) under indicated conditions containing M-CSF (50 ng/mL) and recombinant soluble RANKL (25 ng/mL, PeproTech Ltd.) with or without latent-TGFβ1 (10 ng/ml, R & D Systems), TGFβ1 (0.016–10 ng/ml, R & D Systems), TGFβ3 (0.016–10 ng/ml, R & D Systems) or BMP2 (40–1,000 ng/ml, Pepro Tech Ltd.). Medium was changed every 2 days. Osteoclastogenesis was evaluated by TRAP staining, and TRAP-positive multi-nuclear cells containing more than three nuclei were scored as osteoclasts.

For some experiments, supernatants from osteoclast culture for five days with or without latent-TGFβ1-Fc (10 μg/ml, R & D Systems) were added to secondary cultures, and osteoclastogenesis was evaluated by TRAP staining or expression of osteoclastic genes.

### Quantitative PCR analysis

Total RNAs were isolated from bone marrow cultures using TRIzol reagent (Invitrogen Corp.), and cDNA synthesis was performed using oligo(dT) primers and reverse transcriptase (Wako Pure Chemicals Industries). Quantitative PCR was performed using SYBR Premix ExTaq II reagent and a DICE Thermal cycler (Takara Bio Inc.), according to the manufacturer’s instructions. *β-actin* (*Actb*) expression served as an internal control. Primers used for realtime PCR analysis were as follows.

*β-actin*-forward: 5′-TGAGAGGGAAATCGTGCGTGAC-3′

*β-actin*-reverse: 5′-AAGAAGGAAGGCTGGAAAAGAG-3′

*Ctsk*-forward: 5′-ACGGAGGCATTGACTCTGAAGATG-3′

*Ctsk*-reverse: 5′-GGAAGCACCAACGAGAGGAGAAAT-3′

*NFATc1*-forward: 5′-CAAGTCTCACCACAGGGCTCACTA-3′

*NFATc1*-reverse: 5′-GCGTGAGAGGTTCATTCTCCAAGT-3′

*Smad4*-forward: 5′-TATCACTATGAGCGGGTTGTCTCA-3′

*Smad4*-reverse: 5′-TCAAAATCTGGGCTCTTGTTCAG-3′

*Prdm1*-forward: 5′-TTCTTGTGTGGTATTGTCGGGACTT-3′

*Prdm1*-reverse: 5′-TTGGGGACACTCTTTGGGTAGAGTT-3′

*Bcl6*-forward: 5′-AGACGCACAGTGACAAACCATACAA-3′

*Bcl6*-reverse: 5′-GCTCCACAAATGTTACAGCGATAGG-3′

*Irf8*-forward: 5′-CAGGATTACAATCAGGAGGTGGA-3′

*Irf8*-reverse: 5′-AATCGAATGTCCTTCAGTGGGTAA-3′

*BMP2*-forward: 5′-CTAGATCTGTACCGCAGGCACT-3′

*BMP2*-reverse: 5′-TTTTCCCACTCATCTCTGGAAG-3′

*TGFβ1*-forward: 5′-GACCCTGGATACCAACTATTGC-3′

*TGFβ1*-reverse: 5′-CAGACAGAAGTTGCCATGGTAGC-3′

*TGFβ2*-forward: 5′-ATGAACCCAAAGGGTACAATGCT-3′

*TGFβ2*-reverse: 5′-AGCTTCGGGATTTATGGTGTTGT-3′

*TGFβ3*-forward: 5′-CCCTGGACACCAATTACTGCTTC-3′

*TGFβ3*-reverse: 5′-GCCTGAGCAGAAGTTGGCATAGT-3′

### Western blot analysis

Whole cell lysates were prepared from 8-week-old *Smad4*^*f/f*^ (control) or *Ctsk*^*cre/*+^*Smad4*^*f/f*^ mice bone marrow cultures using RIPA buffer (1% Tween 20, 0.1% SDS, 150 mM NaCl, 10 mM Tris-HCl (pH 7.4), 0.25 mM phenylmethylsulfonylfluoride, 10 μg/mL aprotinin, 10 μg/mL leupeptin, 1 mM Na3VO4, 5 mM NaF (Sigma-Aldrich Co.)). Equivalent amounts of protein were separated by SDS-PAGE and transferred to a PVDF membrane (EMD Millipore Corp.). Proteins were detected by using anti-Smad4 (9515, Cell Signaling) or anti-Actin (Sigma**-**Aldrich Co., St Louis, MO) antibody.

### Chromatin immune precipitation sequence (ChIP seq) assay

Osteoclasts cultured with M-CSF + RANKL + TGFβ1 were harvested and ChIP-seq assay performed using anti-Smad2/3 antibody (BD biosciences, San Jose, CA, USA) as described^37^.

### *In vivo* osteolysis model

100 μl of PBS containing LPS (50 mg/kg) was injected with or without latent-TGFβ1-Fc onto the periosteal surface of calvariae in living 8-week old wild-type mice. Five days later, mice were euthanized, and calvariae and spleen were harvested for micro-computed tomography (micro-CT) and flow cytometry, respectively. Micro-CT was performed using a (micro-CT) scan R_mCT2 system (Rigaku Corp., Tokyo, Japan). For flow cytometry, spleen cells were stained with anti-CD4 and anti-IL-17 antibodies, and analyzed by FACSCanto™ II (BD Biosciences, San Jose, CA, USA) as described^55^. Spleen cells were collected from each group.

### Immunofluorescent staining

Surgical sections of calvaria were stained with mouse anti-Cathepsin K (Ctsk) (1:100 Daiichi Finechemical Co., Toyama, Japan) and anti-NFATc1 (NFATc1) (1:00 Santa Cruz Biotechnology) followed by Alexa488-conjugated goat anti-mouse Ig’ (1:200; Invitrogen, Carlsbad, CA). DAPI (1:750; Wako Pure Chemicals Industries, Osaka, Japan) was used for a nuclear stain.

### Statistical analysis

Results are expressed as the mean ± s.d. Differences between groups were examined for statistical significance using Student *t* test.

## Additional Information

**How to cite this article**: Morita, M. *et al*. Smad4 is required to inhibit osteoclastogenesis and maintain bone mass. *Sci. Rep*. **6**, 35221; doi: 10.1038/srep35221 (2016).

## Supplementary Material

Supplementary Information
