# Supplementary material for: Smad4 is required to inhibit osteoclastogenesis and maintain bone mass
Source: Sci Rep. 2016 Oct 12;6:35221. doi: 10.1038/srep35221 (PMC5059689; doi:10.1038/srep35221)
Supplement: Supplementary Information [file srep35221-s1.pdf]

Smad4 is required to inhibit osteoclastogenesis and maintain bone mass

Mayu Morita, Shigeyuki Yoshida, Ryotaro Iwasaki, Tetsuro Yasui, Yuiko Sato, Tami Kobayashi, Ryuichi Watanabe, Takatsugu Oike, Kana Miyamoto, Masamichi Takami, Keiko Ozato, Chu-Xia Deng, Hiroyuki Aburatani, Sakae Tanaka, Akihiko Yoshimura, Yoshiaki Toyama, Morio Matsumoto, Masaya Nakamura, Hiromasa Kawana, Taneaki Nakagawa and Takeshi Miyamoto

Supplementary Figure Legends

Figure S1 Both TGFβ1 and TGFβ3 stimulate osteoclastogenesis in Raw264.7 cells. Total RNA was isolated from Raw264.7 cells cultured in the presence of RANKL with or without TGFβ1 or TGFβ3 (10 ng/ml each), and *Ctsk* and *NFATc1* expression was analyzed by realtime PCR. Data represent mean *Ctsk* or *NFATc1* expression relative to *β-actin* ± SD (n=3). **P<0.01; ***P<0.001; NS, not significant. Representative data of at least two independent experiments are shown.

Figure S2 Osteoclast formation is suppressed by inhibition of TGFβ signaling by SB431542. (**a-c**) Osteoclast progenitors from wild-type mice were cultured with or without SB431542 (SB) in the presence or absence of M-CSF (M) and RANKL (R). Osteoclast formation was evaluated by TRAP staining (**a**), by the number of multi-nuclear TRAP-positive cells (**b**) and by *Ctsk* and *NFATc1* expression as analyzed by realtime PCR. (**c**) Data represent mean *Ctsk* or *NFATc1* expression relative to *β-actin* ± SD (n=3). Bar=100 µm. ***P<0.001. Representative data of at least two independent experiments are shown.

Figure S3 Osteoclast formation is promoted or inhibited by TGFβ1 in a concentration-dependent manner. Osteoclast progenitors from wild-type mice were cultured with or without the indicated concentrations of TGFβ1 in the presence or absence of M-CSF (M) and RANKL (R). Osteoclast formation was evaluated by TRAP

staining (**a**), by the number of multi-nuclear TRAP-positive cells (**b**) and by *Ctsk* and *NFATc1* expression as analyzed by realtime PCR (**c**). Data represent mean *Ctsk* or *NFATc1* expression relative to *β-actin* ± SD (*n*=3). Bar=100 μm. *P<0.05; **P<0.01; ***P<0.001; NS, not significant. Representative data of at least three independent experiments are shown.

Figure S4 Osteoclast formation is promoted or inhibited by TGFβ3 in a concentration-dependent manner. Osteoclast progenitors from wild-type mice were cultured with or without the indicated concentrations of TGFβ3 in the presence or absence of M-CSF (M) and RANKL (R). Osteoclast formation was evaluated by TRAP staining (**a**), by the number of multi-nuclear TRAP-positive cells (**b**) and by *Ctsk* and *NFATc1* expression as analyzed by realtime PCR. (**c**) Data represent mean *Ctsk* or *NFATc1* expression relative to *β-actin* ± SD (*n*=3). Bar=100 μm. *P<0.05; **P<0.01; ***P<0.001; NS, not significant. Representative data of at least three independent experiments are shown.

Figure S5 Osteoclast formation is promoted then inhibited by increasing concentration of BMP2. Osteoclast progenitors from wild-type mice were cultured with or without indicated concentrations of BMP2 in the presence or absence of M-CSF (M) and/or RANKL (R). Osteoclast formation was evaluated by TRAP staining (**a**) and by the number of multi-nuclear TRAP-positive cells (**b**). Bar=100 μm. *P<0.05; **P<0.01. Representative data of at least three independent experiments are shown.

Figure S6 High TGFβ3 concentrations inhibit osteoclastogenesis via Smad4 activity. Osteoclast progenitors from control (*Smad4*$^{flox/flox\ or\ flox/+}$) or Smad4 cKO mice were cultured with or without 10 ng/ml TGFβ3 in the presence or absence of M-CSF (M) and RANKL (R). Osteoclast formation was evaluated by TRAP staining (**a**), by the number of multi-nuclear TRAP-positive cells (**b**) and by *Ctsk* and *NFATc1* expression as analyzed by realtime PCR (**c**). Data represent mean *Ctsk* or *NFATc1* expression relative to *β-actin* ± SD (*n*=3). Bar=100 μm. **P*<0.05; ***P*<0.01; ****P*<0.001; NS, not significant. Representative data of at least three independent experiments are shown.

Figure S7 TGFβ3 stimulates *Bcl6* and *Irf8* expression in osteoclasts via Smad4. Osteoclast progenitors were isolated from wild-type, Bcl6-deficient (**a-c**) or Irf8-deficient (**d-f**) mice and cultured in the presence or absence of M-CSF (M) and RANKL (R), with or without 10 ng/ml TGFβ3. Osteoclast formation was evaluated by TRAP staining (**a** and **d**), by the number of multi-nuclear TRAP-positive cells (**b** and **e**) and by *Ctsk* and *NFATc1* expression as analyzed by realtime PCR (**c** and **f**). Data represent mean *Ctsk* or *NFATc1* expression relative to *β-actin* ± SD (*n*=3). Bar=100 μm. **P*<0.05; ***P*<0.01; ****P*<0.001; NS, not significant. Representative data of at least three independent experiments are shown.

Figure S8 High TGFβ3 inhibits *Prdm1* expression in osteoclasts via Smad4. Osteoclast progenitors from control (*Smad4*$^{flox/flox\ or\ flox/+}$) or Smad4 cKO mice were

cultured with or without 10 ng/ml TGFβ3 in the presence or absence of M-CSF and RANKL. *Prdm1* expression was analyzed by realtime PCR. Data represent mean *Prdm1* expression relative to *β-actin* ± SD (*n*=3). ***P<0.001; NS, not significant. Representative data of at least two independent experiments are shown.

Figure S9 *Prdm1* expression is upregulated by either TGFβ1 or TGFβ3 in Raw264.7 cells. Total RNA was isolated from Raw264.7 cells cultured in the presence of RANKL with or without TGFβ1 or TGFβ3 (10 ng/ml each). *Prdm1, Bcl6* and *Irf8* expression was analyzed by realtime PCR. Data represent mean transcript expression relative to *β-actin* ± SD (*n*=3). *P<0.05; **P<0.01; ***P<0.001; NS, not significant. Representative data of at least two independent experiments are shown.

Figure S10 *Prdm1* is upregulated in wild-type osteoclasts treated with BMP2. Total RNA was isolated from wild-type osteoclasts cultured in the presence or absence of M-CSF (M) and RANKL (R) with or without BMP2 (200ng/ml). *Prdm1, Bcl6* and *Irf8* expression was analyzed by realtime PCR. Data represent mean transcript expression relative to *β-actin* ± SD (*n*=3). Bar=100 μm. **P<0.01; ***P<0.001. Representative data of at least two independent experiments are shown.

Figure S11 *Prdm1* expression is inhibited, while *Bcl6* and *Irf8* are upregulated by SB431542 treatment. Total RNA was isolated from wild-type osteoclasts cultured in the presence or absence of M-CSF (M) and RANKL (R) with or without the TGFβ

inhibitor SB431542 (SB). *Prdm1, Bcl6* and *Irf8* expression was analyzed by realtime PCR. Data represent mean transcript expression relative to *β-actin* ± SD (n=3). Bar=100 μm. **P<0.01; ***P<0.001. Representative data of at least three independent experiments are shown.

Figure S12 A schematic model showing the roles of TGFβ1 and TGFβ3 in bone remodeling. (**a**) Latent-TGFβ1 accumulates in bone matrix and is activated by osteoclastic bone-resorption. Osteoclast activity creates an acidic environment (H^+) to convert latent-TGFβ1 to an active form. Activated TGFβ1 stimulates bone formation via Smad4 activity. (**b**) Activated TGFβ1 also acts to inhibit osteoclastogenesis via Smad4 in a negative feedback loop, and Smad4-dependent osteoclast inhibition is required to maintain bone mass. (**c**) Boxed area in panel (**b**) is shown in panel (**c**). Smad4 is required to inhibit *Prdm1* followed by *Irf8* and *Bcl6* upregulation to block osteoclastogenesis.

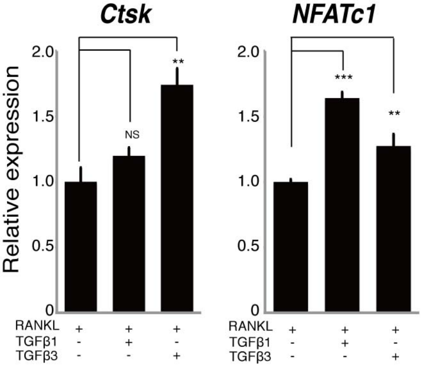

Ctsk
NFATc1
Relative expression
2.0
1.5
1.0
0.5
0
NS
**
***
**
RANKL + + +
TGFβ1 - + -
TGFβ3 - - +
RANKL + + +
TGFβ1 - + -
TGFβ3 - - +

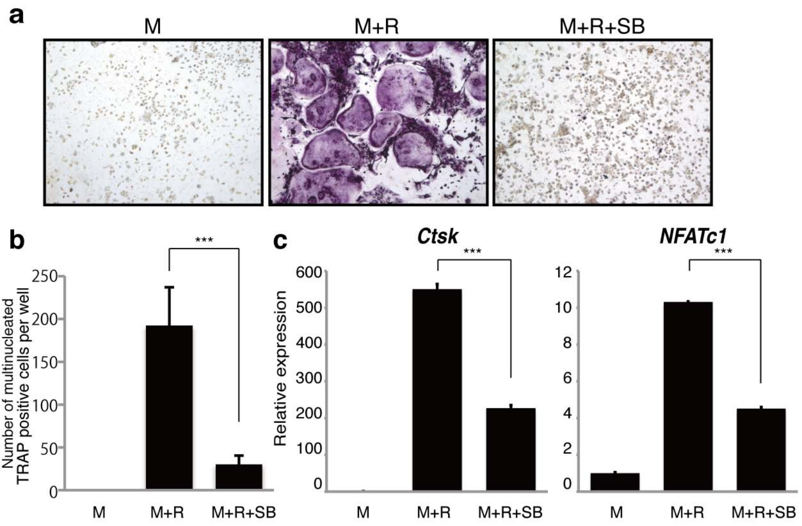

a
M
M+R
M+R+SB
b
***
Number of multinucleated TRAP positive cells per well
250
200
150
100
50
0
M
M+R
M+R+SB
c
Ctsk
***
Relative expression
600
500
400
300
200
100
0
M
M+R
M+R+SB
NFATc1
***
12
10
8
6
4
2
0
M
M+R
M+R+SB

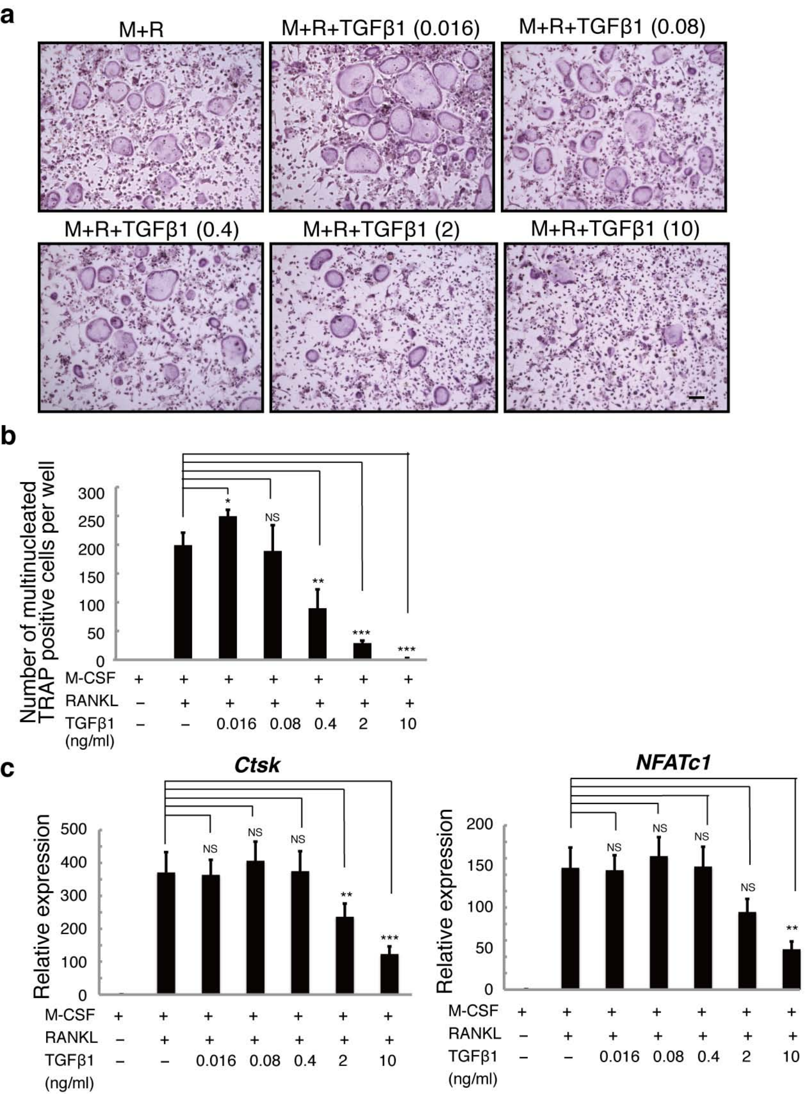

a
M+R
M+R+TGFβ1 (0.016)
M+R+TGFβ1 (0.08)
M+R+TGFβ1 (0.4)
M+R+TGFβ1 (2)
M+R+TGFβ1 (10)
b
Number of multinucleated TRAP positive cells per well
300
250
200
150
100
50
0
*
NS
**
***
***
M-CSF + + + + + + +
RANKL – + + + + + +
TGFβ1 (ng/ml) – – 0.016 0.08 0.4 2 10
c
Ctsk
Relative expression
500
400
300
200
100
0
NS
NS
NS
**
***
M-CSF + + + + + + +
RANKL – + + + + + +
TGFβ1 (ng/ml) – – 0.016 0.08 0.4 2 10
NFATc1
Relative expression
200
150
100
50
0
NS
NS
NS
NS
**
M-CSF + + + + + + +
RANKL – + + + + + +
TGFβ1 (ng/ml) – – 0.016 0.08 0.4 2 10

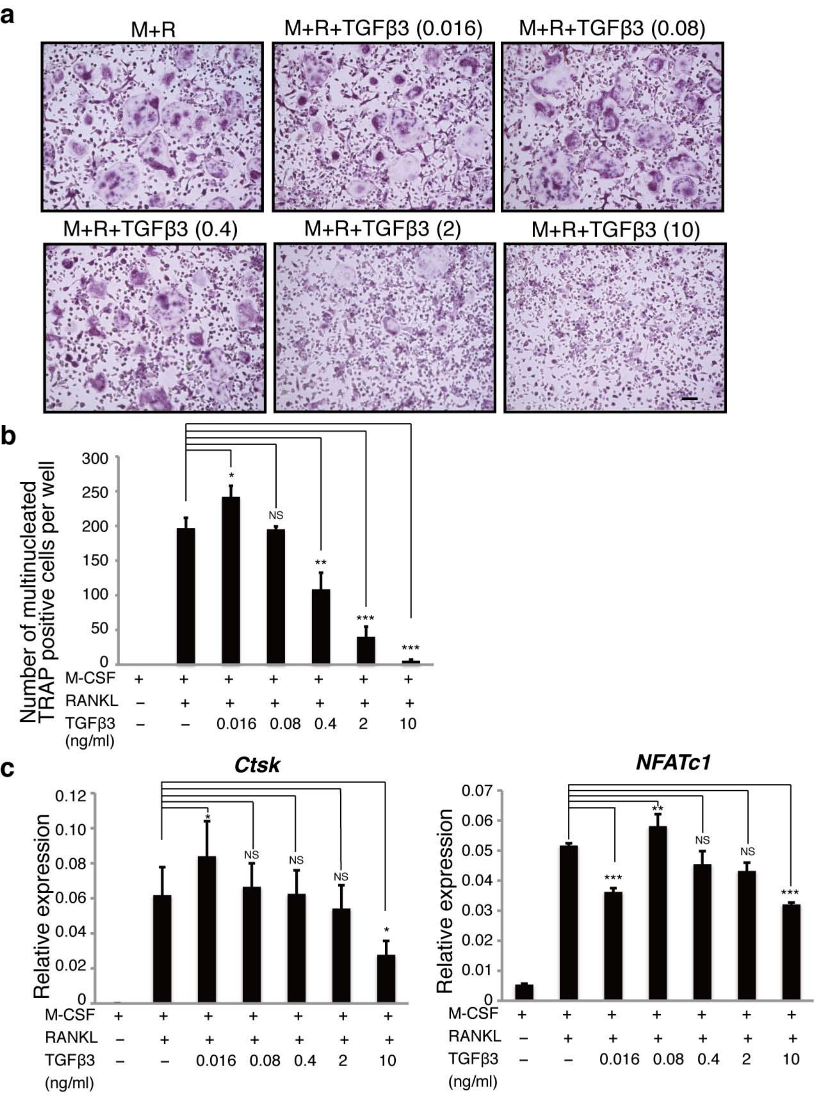

a
M+R
M+R+TGFβ3 (0.016)
M+R+TGFβ3 (0.08)
M+R+TGFβ3 (0.4)
M+R+TGFβ3 (2)
M+R+TGFβ3 (10)
b
Number of multinucleated TRAP positive cells per well
300
250
200
150
100
50
0
*
NS
**
***
***
M-CSF + + + + + + +
RANKL – + + + + + +
TGFβ3 (ng/ml) – – 0.016 0.08 0.4 2 10
c
Ctsk
Relative expression
0.12
0.10
0.08
0.06
0.04
0.02
0
*
NS
NS
NS
*
M-CSF + + + + + + +
RANKL – + + + + + +
TGFβ3 (ng/ml) – – 0.016 0.08 0.4 2 10
NFATc1
Relative expression
0.07
0.06
0.05
0.04
0.03
0.02
0.01
0
***
**
NS
NS
***
M-CSF + + + + + + +
RANKL – + + + + + +
TGFβ3 (ng/ml) – – 0.016 0.08 0.4 2 10

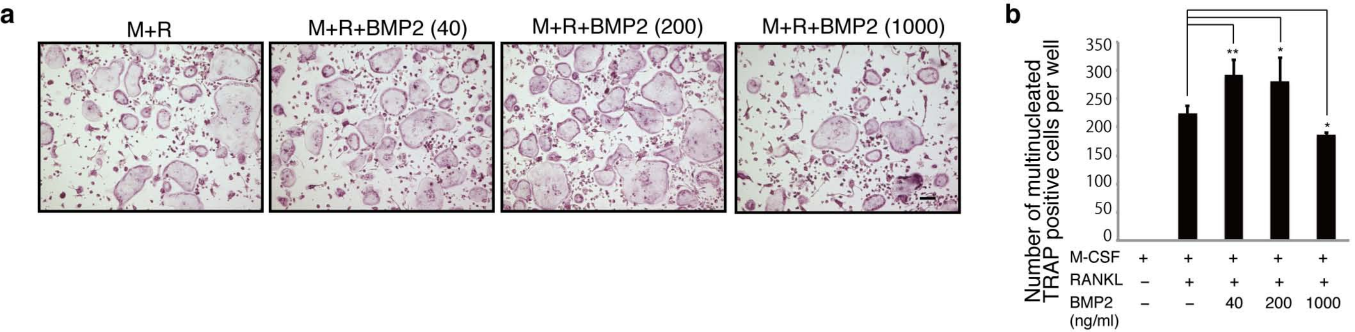

a
M+R
M+R+BMP2 (40)
M+R+BMP2 (200)
M+R+BMP2 (1000)
b
Number of multinucleated TRAP positive cells per well
350
300
250
200
150
100
50
0
**
*
*
M-CSF + + + + +
RANKL – + + + +
BMP2 (ng/ml) – – 40 200 1000

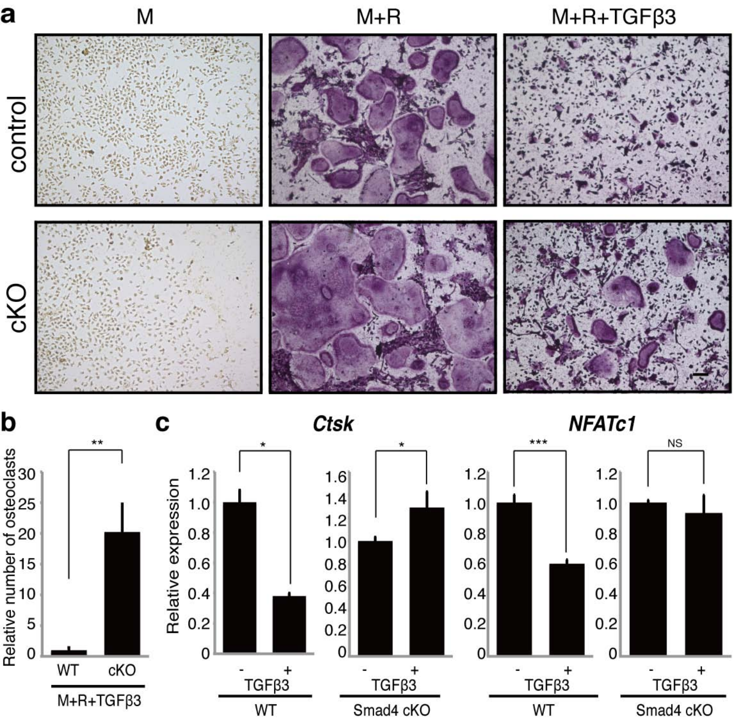

a
M
M+R
M+R+TGFβ3
control
cKO
b
Relative number of osteoclasts
**
30
25
20
15
10
5
0
WT
cKO
M+R+TGFβ3
c
Ctsk
NFATc1
Relative expression
*
*
***
NS
1.2
1.0
0.8
0.6
0.4
0.2
0
1.6
1.4
-
+
TGFβ3
WT
Smad4 cKO

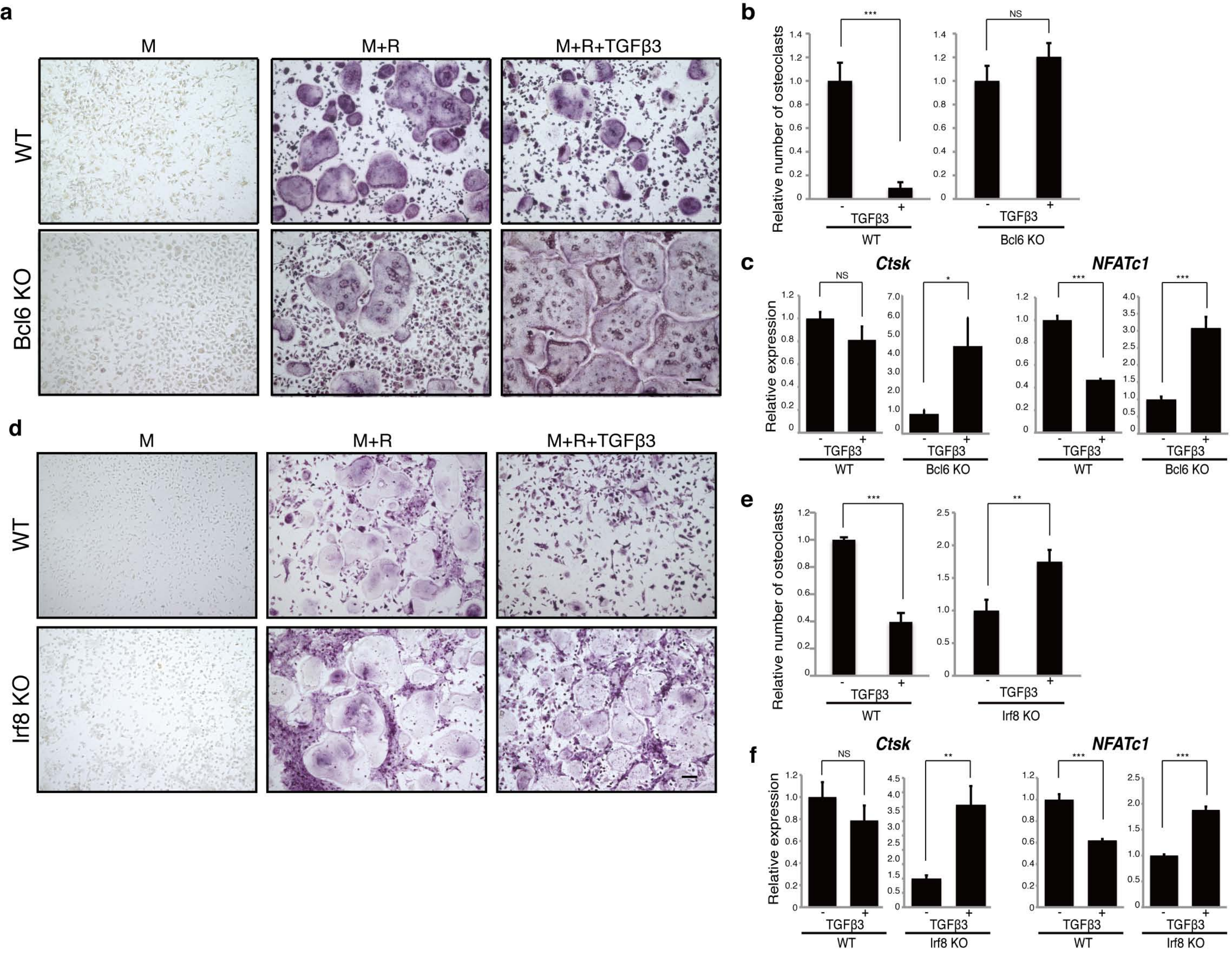

a
M
M+R
M+R+TGFβ3
WT
Bcl6 KO
b
Relative number of osteoclasts
TGFβ3
WT
Bcl6 KO
***
NS
c
Ctsk
NFATc1
Relative expression
TGFβ3
WT
Bcl6 KO
NS
*
***
***
d
M
M+R
M+R+TGFβ3
WT
Irf8 KO
e
Relative number of osteoclasts
TGFβ3
WT
Irf8 KO
***
**
f
Ctsk
NFATc1
Relative expression
TGFβ3
WT
Irf8 KO
NS
**
***
***

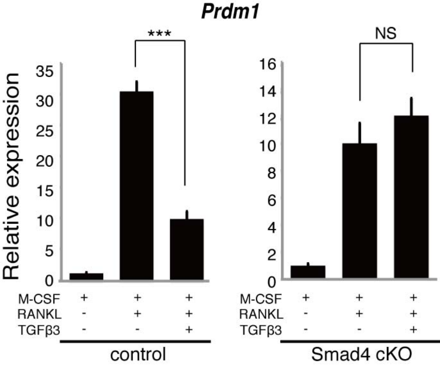

Prdm1
***
NS
Relative expression
0
5
10
15
20
25
30
35
0
2
4
6
8
10
12
14
16
M-CSF + + +
RANKL - + +
TGFβ3 - - +
control
M-CSF + + +
RANKL - + +
TGFβ3 - - +
Smad4 cKO

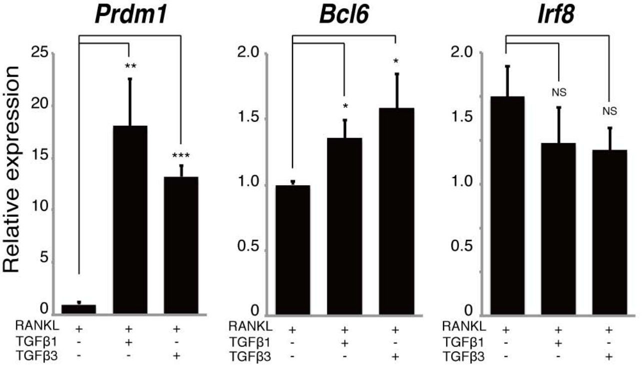

Prdm1
Bcl6
Irf8
Relative expression
25
20
15
10
5
0
**
***
2.0
1.5
1.0
0.5
0
*
*
NS
NS
RANKL + + +
TGFβ1 - + -
TGFβ3 - - +

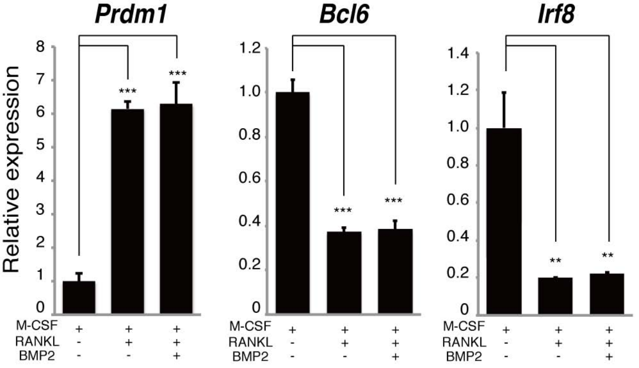

Prdm1
Bcl6
Irf8
Relative expression
***
***
***
***
**
**
M-CSF
RANKL
BMP2

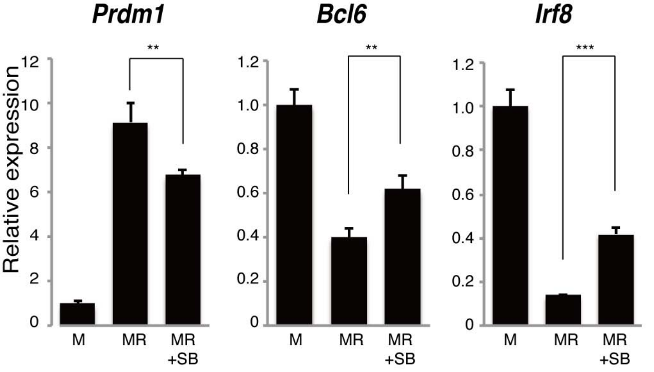

Prdm1
Bcl6
Irf8
Relative expression
**
**
***
0
2
4
6
8
10
12
0
0.2
0.4
0.6
0.8
1.0
1.2
M
MR
MR
+SB

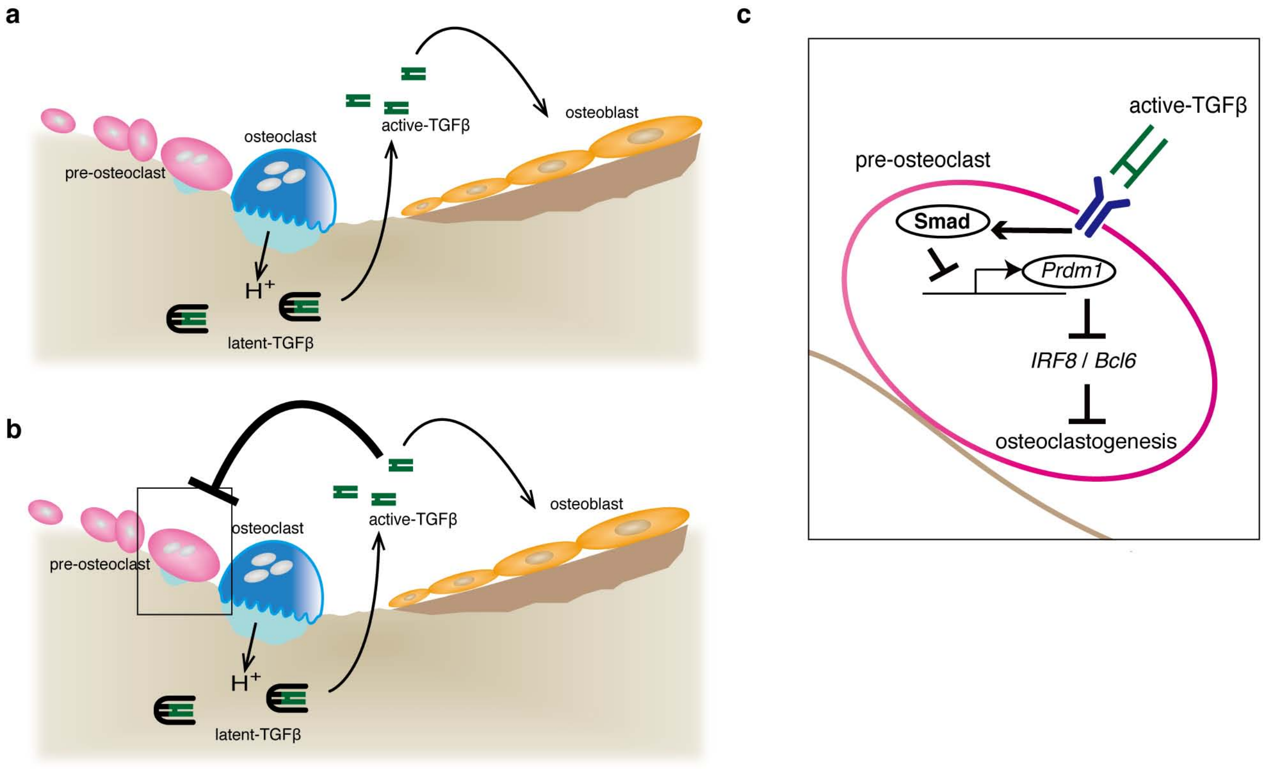
a
b
c
osteoclast
pre-osteoclast
active-TGFβ
osteoblast
H+
latent-TGFβ
osteoclast
pre-osteoclast
active-TGFβ
osteoblast
H+
latent-TGFβ
active-TGFβ
pre-osteoclast
Smad
Prdm1
IRF8 / Bcl6
osteoclastogenesis
